# Early Childhood Caries in Obese Children: The Status and Associated Factors in the Suburban Areas in Hanoi, Vietnam

**DOI:** 10.3390/ijerph18168844

**Published:** 2021-08-22

**Authors:** Ha Van Hung, Vo Truong Nhu Ngoc, Hue Vu Thi, Dinh-Toi Chu

**Affiliations:** 1 School of Odonto Stomatology, Hanoi Medical University, Hanoi 100000, Vietnam; drhahungtb@gmail.com; 2 Center for Biomedicine and Community Health, International School, Vietnam National University, Hanoi 100000, Vietnam; vuhueytcchmu@gmail.com; 3 Department of Cell Biology and Anatomy, College of Medicine, National Cheng Kung University, Tainan 70101, Taiwan

**Keywords:** obese children, early childhood caries, like soft drinks, drink milk at night, drink milk at night regularly

## Abstract

Obesity and early childhood caries are two prominent health problems affecting the majority of children worldwide. Thus, early childhood caries in obese children must be studied. This study was conducted to investigate the status of early childhood caries in obese children in Hanoi, Vietnam, and its associated factors. A cross-sectional study was conducted on 234 obese children, 234 normal children (non-obese) aged 36 to 71 months, and their mothers at some kindergartens in Hanoi. Study subjects were randomly selected with similarities in age, gender, and study location. Decayed tooth of children was detected by clinical examination and Diagnodent Kavo 2190 machine of Germany. In addition, a questionnaire for their mothers was used to find out related factors. We found that, in the Obese Group, the rate of early childhood caries (ECC), severe-early childhood caries (S-ECC), dmft index (the number of decayed teeth, teeth lost due to cavities, filled decayed teeth or filled cavity), and dmfs index (the number of surfaces of the teeth decay, surfaces of teeth were lost due to cavities, surfaces of filled decayed teeth) were 82.91%, 59.83%, 6.84 ± 4.92, and 9.10 ± 7.48, respectively. In the Normal Group, these rates were smaller than in the Obese Group, but the difference was not statistically significant. Regarding related factors, the hobby of drinking soft drinks, the habits and frequency of drinking milk at night and eating sweet marshmallows were associated with ECC in the Obese Group with *p* < 0.05. In conclusion, the higher rates of ECC were seen in obese children, with eating hobbies and habits being the related factors. Therefore, it is necessary to have appropriate policies and effective communication strategies to minimize ECC in the future.

## 1. Introduction

Obesity and early childhood caries (ECC) are two prominent health problems affecting the majority of children worldwide [1]. Instead of showing signs of decreasing, obesity in children had increased dramatically from 2000 to 2019. The percentage of obese children under the age of five increased from 4.9% to 5.6% in the world [2]. This percentage has doubled in Southeast Asia from 3.2% to 7.5%, and in Vietnam from 2.6% to 5.9% [3]. ECC is one or more damage of the tooth due to tooth decay, or tooth loss due to decay or filled cavities using fillings on any milk tooth in babies between birth and 71 months of age [4]. The severe early childhood caries (S-ECC) is the presence of any sign of smooth-surface caries in children < 3 years of age; 1 or more cavity teeth are filled in children from ages 3 through 5; or dmft ≥4 for age 3, ≥5 for age 4, or ≥6 for age 5 [4]. The rate of ECC in the world is about 60–90%, with the highest in Asia and Latin America [5]. In Vietnam, Nguyen YHT studies showed that ECC in children 3–5 years old accounted for 79.1% [6], and this rate of 4 years old children in Vo Truong Nhu Ngoc’s study was 92% [7]. In addition, many studies have shown that the rate of ECC, S-ECC, dmft, and dmfs index in obese children was higher than in normal children [8,9,10]. In contrary, some studies have shown that the rate of tooth decay in obese children was lower than in normal or malnourished children [11].

Several studies on the correlation between obesity and ECC have been conducted, although their results have shown some inconsistencies. Matina et al. conducted a review study of twenty-one studies, including nine studies on the correlation between tooth decay and obesity [12]. However, obesity and tooth decay are multi-factor chronic diseases [13], including fundamental causes and some related factors such as nutrition, snacking habits, preferences and habits of eating, oral hygiene habits, etc. [14,15,16]. This study aims to describe the situation of ECC in obese children in Hanoi, Vietnam, and its associated factors.

## 2. Methods

### 2.1. Research Subjects

A total of 234 obese children, 234 normal children from 36 to 71 months, and their mothers were selected to participate in this research.

### 2.2. Data Collection Method

Time and place of research:

+ Data collection time: From September 2019 to June 2020.
+ Place of the study: Some kindergartens of 4 suburban districts in Hanoi (Phu Xuyen district, My Duc district, Hoai Duc district, and Thuong Tin district).

Research design: The cross-sectional study was conducted

Sample size:

(1) $$n=Z_{\left(1-\alpha /2\right)}^{2}\frac{p\left(1-p\right)}{d^{2}}$$

*n*: Sample size.

*Z*_1−*α*/2_ = 1.96 for 95% confidence interval.

*p* = 0.8 based on early childhood caries rate is 80% of the Farsi DJ’s study [11];

Delta (*d*): Error occurred in data selection (*d* = 0.055).

From the formula, the sample size has been calculated (*n* = 234 children).

Sample selection: Studied subjects were selected randomly with similarities in age, gender, and study location. The estimated obesity rate is 5% [3]. We clinically examined 6000 children from 30 kindergartens of 4 districts in Hanoi until 234 obese children and 234 normal children were selected. Group classification was based on body mass index (BMI). Based on Barlow and experts’ proposed cut-off points, Al Herbish classified BMI by age and gender: underweight with <5th percentile, normal with 5th–84th percentile, overweight with 85th–94th percentile and obese with >95th percentile [11]. The estimated prevalence of obesity was 5%, as we checked the weight of 6000 children from 30 kindergartens in 4 districts of Hanoi city until we got enough 234 obese children.

Selection criteria: The age range of the children was from 36 to 71 months. It contained healthy children with no maxillofacial deformities. The children had a cooperative attitude in the research process. The children’s mothers agreed to participate in the interviews.

Exclusion criteria: Children or their mothers give up at any stage in the research.

Data collection: Clinical examination by direct observation. Classification of caries according to the International Caries Detection and Assessment System II (ICDAS II) [17]. This system has 6 different classification codes: 0—sound; 1—first visual change in enamel; 2—distinct visual change in enamel; 3—localized enamel breakdown; 4—underlying dentine shadow; 5—distinct cavity with visible dentine; and 6—extensive cavity with visible dentine. We checked back with Dianogdent (DD) KaVo 2190 (KaVo, Biberach, Germany) fluorescent laser equipment. This device can detect back-scattered fluorescence from teeth through a sensor that emits 655 nm monochromatic light [18]. The DD scores are divided into three levels with ranges from 0–99: 0–13: healthy; 14–30: deep enamel damage, and >30: deep dentin damage. The caries experience is described based on two indexes: dmft (the number of decayed teeth, teeth lost due to cavities, filled decayed teeth or filled cavity), and dmfs (the number of surfaces of the teeth decay, surfaces of teeth were lost due to cavities, surfaces of filled decayed teeth). During the examination, we use dental examination mirror, smartphones to monitor and save images. We used questionnaires to interview mothers about related factors.

### 2.3. Statistical Analysis

Data was entered into the Epidata software. SPSS 20 (IBM Corp, Armonk, New York, USA) software was used for data analysis. *T*-test, ANOVA test, univariate and multivariate regression were used to analyze the correlation between ECC and obesity. After analyzing univariate regression, multivariate regression analysis was conducted in 2 stages: Stage 1: using backward-Wald variable input method with variables with *p* < 0.1 for each group (total, obese and normal); Stage 2: using enter variable input method with the selected variables in stage 1.

## 3. Results

In the Obese Group, the rate of ECC was 82.91%, in which, male accounts for 50% and female accounts for 32.91%. The rate of S-ECCC was 59.8%; the dmft index was 6.84 ± 4.92; the dmfs index was 9.10 ± 7.48. In the Normal Group, these rates were 82.05%, 44.87%, 37.18%, 64.96%, 6.11 ± 4.16, 8.49 ± 6.68, respectively. These rates were smaller than that of the Obese Group, but the difference was not statistically significant with *p* ≥ 0.05 (Table 1).

The results in Table 2 showed that children who liked soft drinks had a 2.38 times higher risk of ECC than children who did not like them in the Obese Group with *p* = 0.016. However, in the Normal Group, there was no statistically significant difference. Similarly, children who liked sweet candy had a 2.82 times higher risk of ECC than children who did not like it in the Obese Group with *p* = 0.013, and there was no statistically significant difference in the Normal Group. Eating sweet candy and cake regularly had a 3.5 times higher risk of ECC than rarely consumed in the Obese Group with *p* = 0.003. Children who drank milk at night had a rate of ECC 2.1 times higher than children who did not drink, and 4 times higher risk of ECC than who rarely drank in the Obese Group. The rate of ECC in children who liked snacking was 5 times higher than those who did not like snacking in the Obese Group, and 2 times higher in the Normal Group. Children who were only given periodic oral evaluation when they had a toothache had a 4.6 times higher risk of ECC than those who were evaluated 6 months/time in the Obese Group, and there was no statistically significant difference in the Normal Group.

The rate of ECC in children who liked soft drinks was 3.35 times higher than children who did not like with *p* < 0.01. Similarly, eating sweet marshmallows had a 3.16 times higher risk of ECC. Periodic oral evaluation when a child had a toothache had a 3.93 times higher risk than 6 months/time. The number of mothers who perceived the need for ECC treatment was 3.13 times higher than the number of mothers who did not perceive. Especially, the rate of ECC in children who liked eating sticky sweet cake was 13.20 times higher than that of children who did not like it. On the contrary, children who had no interest in eating sweet candy and cake has a 3.93 times higher risk of ECC than children who had (Table 3).

In the Obese Group, the rate of ECC in children who liked soft drinks was 4.14 times higher than that of children who are not fond of it. Drinking milk at night had a 4.22 times higher risk of ECC and eating sweet marshmallows had a 12.5 times higher risk of ECC with *p* < 0.001. In the Normal Group, the rate of ECC who relished sticky sweet cake was 15 times higher than that of children who are not interested in it. Eating hard lollipop and cleaning teeth after snacking were two protect factors against ECC with *p* < 0.001 (Table 4).

## 4. Discussion

Our cross-sectional study in 234 obese children showed that the rate of ECC was 82.91%. This result was similar to those of Deema J. Farsi’s study and Juárez-López ML’s study. Deema J. Farsi conducted a research on 915 children and the rate of ECC was 80% [11]. Similarly, this rate in the Juárez-López ML’s was 79% [19]. In the Normal Group, the rate of ECC in our study was 82.05%, which was higher than that of some studies in Vietnam such as Ngo Khanh Linh’s study (75%) [20], and Vu Manh Tuan’s study (74%) [21]. The difference can be explained that our study used the Diagnodent Kavo 2190 machine of Germany. Therefore, more ECC cases might have been detected. On the contrary, this rate was lower than that of Vo Truong Nhu Ngoc’s study (92.5%). This can be explained by the location of the study, as Vo Truong Nhu Ngoc’s study was conducted in rural areas and tap water in that area had not been fluorination. Our study showed that the rate of ECC in obese children was higher than that in normal children, but the difference was not significant. This result was different from the result from the study of Deema J. Farsi et al. when they showed that the rate of ECC in obese children was 80%, lower than that of normal and underweight children (87%) with *p* = 0.012 [11]. The dmft and dmfs indexes in the Obese Group were 6.84 ± 4.92 and 9.10 ± 7.48, respectively higher than that in the Normal Group, but the difference was not statistically substantial. Pikramenou V researched 2180 children and the dmfs index in obese children was 1.99 times higher than that in normal and overweight children. Similarly, Angelopoulou MV et al. showed that dmft and dmfs index were higher in normal, overweight, or malnourished children than those in obese children [12]. This may be due to the differences in studied subjects, locations (Pikramenou V’s study was conducted in suburban areas), and the economy of Vietnam.

Obesity and ECC are multi-factor chronic diseases [13], including fundamental causes and some related factors such as nutrition, snacking habits, preferences and habits of eating, oral hygiene habits, etc. Our results showed that the rate of ECC in obese children was associated with using soft drinks, drinking milk at night, and eating sweet marshmallows. In the Normal Group, the rate of ECC of children who liked sticky sweet cake was 15 times higher than that of children who did not like it. M Costacurta also showed that using sugary drinks, frequency of sugar consumption were risk factors for obesity and ECC [14]. Another study also showed that children under five years old were at greater risk of ECC if they drink soft drinks (OR = 1.26), eat cakes and chocolate regularly (OR = 1.56) [15]. In addition, high sugar intake was associated with an increase in dmft index with OR = 0.32; 95% CI: 0.06–0.58 [16]. Whole milk has a protective effect on the teeth against tooth enamel, limits the progression of caries [22]. When milk is added with sugar, the role of protecting tooth enamel decreases, which leads to the same risk of tooth decay as some sugary drinks [22]. Commercial milk is often added with extra sugar, especially baby milk. Some studies showed that the rate of ECC in children who drank milk with added sugar every day and over 300 mL/day were higher than that of children without this habit [23]. Our result showed that drinking milk at night has a 4.22 times higher risk of ECC, with *p* < 0.001. In addition, Kraljevic et al. reported that children who drank soft drinks at night had a higher dmfs index (30.0 ± 16.23) than that of children who did not (21.5 ± 13.05), with *p* = 0.03 [24]. This shows that night-time drink management plays an important role in ECC prevention.

Our research had some limitations. The number of participants was small. The location of the study was only in suburban districts, so the representativeness might not be for all the study population. We only focus on ECC and obesity. Therefore, we did not examine some type of systemic comorbidity in children such as high blood pressure and diabetes. Furthermore, the association between ECC and suspected etiological factors have not been interpreted with caution, and the causal relationship has not been elucidated. This cross-sectional study provides an overview and a baseline for further studies in the future.

## 5. Conclusions

Our cross-sectional study in 234 obese children showed that the rate of ECC, S-ECC were 82.91% and 59.83%. Dmft and dmfs indexes were 6.84 ± 4.92 and 9.10 ± 7.48, respectively. This rate in obese children was higher than that in normal children, but the difference was not significant. Related factors such as using soft drinks, drinking milk at night, and eating sweet marshmallows were associated with ECC in obese children with *p* < 0.001.

## Figures and Tables

**Table 1:** Caries status in the study subjects.

		Obese Group (*n* = 234)				Normal Group (*n* = 234)				*p*-Value
		Total	Male	Female	*p*-Value	Total	Male	Female	*p*-Value	
**ECC**	**No** *n* (%)	40 (17.09)	21 (8.97)	19 (8.12)	0.361 *	42 (17.95)	22 (9.04)	20 (8.55)	0.786 *	0.808 *
	**Yes** *n* (%)	194 (82.91)	117 (50.00)	77 (32.91)		192 (82.05)	105 (44.87)	87 (37.18)		
**S-ECC**	**No** *n* (%)	94 (40.17)	55 (23.50)	39 (16.67)	0.906 *	82 (35.04)	37 (15.81)	45 (19.23)	0.039 *	0.252 *
	**Yes** *n* (%)	140 (59.83)	83 (35.47)	57 (24.36)		152 (64.96)	90 (38.46)	62 (28.50)		
**Dmft** mean (SD)		6.84 (4.92)	6.99 (5.04)	6.63 (4.75)	0.575 **	6.11 (4.16)	6.31 (4.13)	5.89 (4.22)	0.564 *	0.316 **
**Dmfs** mean (SD)		9.10 (7.48)	9.18 (7.53)	8.99 (7.43)	0.848 **	8.49 (6.68)	8.56 (6.64)	8.05 (6.11)	0.362 *	0.420 **

(* ANOVA test, ** *T*-test: the difference is statistically significant with *p* < 0.05. The numbers in bold indicate a statistically significant difference).

**Table 2:** Related factors in the univariate regression analysis.

Related Factors (*p* < 0.05)	Total (*n* = 468)				Obese Group (*n* = 234)				Normal Group (*n* = 234)			
	No Cavities *n* (%)	Caries *n* (%)	OR (95% CI)	*p*-Value	No Cavities *n* (%)	Caries *n* (%)	OR (95% CI)	*p*-Value	No Cavities *n* (%)	Caries *n* (%)	OR (95% CI)	*p*-Value
**Like soft drinks**												
No	50 (60.98)	182 (47.15)	1		25 (62.50)	80 (41.24)	1		47 (57.32)	80 (52.63)	1	
Yes	32 (39.02)	204 (52.85)	**1.75 (1.08–2.85)**	**0.024**	15 (37.50)	114 (58.76)	**2.38 (1.18–4.79)**	**0.016**	35 (42.68)	72 (47.37)	1.21 (0.70–2.08)	0.493
**Like eating sweet candy**												
No	25 (30.49)	48 (12.44)	1		11 (27.50)	23 (11.86)	1		18 (21.95)	21 (13.82)	1	
Yes	57 (69.51)	338 (87.56)	**3.09 (1.77–5.40)**	**<0.001**	29 (72.50)	171 (88.14)	**2.82 (1.24–6.40)**	**0.013**	64 (78.05)	131 (86.18)	1.75 (0.87–3.52)	0.114
**Sticky sweet cake**												
No	50 (60.98)	50 (12.95)	1		24 (60.00)	24 (12.37)	1		30 (36.59)	22 (14.47)	1	
Yes	32 (39.02)	336 (87.05)	**10.5 (6.16–17.91)**	**<0.001**	16 (40.00)	170 (87.63)	**10.63 (4.95–22.80)**	**<0.001**	52 (63.41)	130 (85.53)	**3.41 (1.80–6.45)**	**0.001**
**Hard lollipop**												
No	43 (52.44)	275 (71.24)	1		20 (50.00)	144 (74.23)	1		46 (56.10)	108 (71.05)	1	
Yes	39 (47.56)	111 (28.76)	**0.45 (0.27–0.72)**	**0.001**	20 (50.00)	50 (25.77)	**2.88 (1.43–5.79)**	**0.003**	36 (43.90)	44 (28.95)	**0.52 (0.30–0.91)**	**0.022**
**Sweet marshmallows**												
No	53 (64.63)	68 (17.62)	1		26 (65.00)	31 (15.98)	1		32 (39.02)	32 (21.05)	1	
Yes	29 (35.37)	318 (82.38)	**8.55 (5.07–14.42)**	**<0.001**	14 (35.00)	163 (84.02)	**9.71 (4.59–20.77)**	**<0.001**	50 (60.98)	120 (78.95)	**2.4 (1.33–4.33)**	**0.004**
**Candy lozenges**												
No	62 (75.61)	207 (53.63)	1		30 (75.00)	106 (54.64)	1		58 (70.73)	75 (49.34)	1	
Yes	20 (24.39)	179 (46.37)	**2.68 (1.55–4.61)**	**<0.001**	10 (25.00)	88 (45.36)	**2.49 (1.15–5.38)**	**0.020**	24 (29.27)	77 (50.66)	**2.48 (1.40–4.40)**	**0.002**
**Frequency of eating sweet candy and cake**												
Regularly	29 (35.37)	215 (55.70)	1		14 (35.00)	115 (59.28)	1		33 (40.24)	82 (53.95)	1	
Rarely	34 (41.46)	86 (22.28)	**0.34 (0.19–0.59)**	**<0.001**	16 (40.00)	38 (19.59)	**0.29 (0.13–0.65)**	**0.003**	25 (30.49)	41 (26.97)	0.66 (0.35–1.25)	0.204
**Drink milk at night**												
No	43 (52.44)	166 (43.01)	1		22 (55.00)	69 (35.57)	1		39 (47.56)	79 (51.97)	1	
Yes	39 (47.56)	220 (56.99)	1.46 (0.91–2.36)	0.120	18 (45.00)	125 (64.43)	**2.21 (1.11–4.41)**	**0.024**	43 (52.44)	73 (48.03)	0.84 (0.49–1.43)	0.520
**Frequency of drinking milk at night**												
Regularly	14 (17.07)	143 (37.05)	1		7 (17.50)	84 (43.30)	1		7 (16.67)	59 (30.73)	1	
Rarely	53 (64.63)	198 (51.30)	**0.37 (0.19–0.68)**	**0.002**	27 (67.50)	85 (43.81)	**0.26 (0.11–0.64)**	**0.003**	26 (61.90)	113 (58.59)	0.52 (0.21–1.26)	0.146
**Like snacking**												
No	39 (47.56)	93 (24.09)	1		19 (47.50)	36 (18.56)	1		32 (39.02)	45 (29.61)	1	
Yes	43 (52.44)	293 (75.91)	**2.85 (1.74–4.67)**	**<0.001**	21 (52.50)	158 (81.44)	**3.97 (1.94–8.15)**	**<0.001**	50 (68.98)	107 (70.39)	1.52 (0.87–2.67)	0.145
**Frequency of snacking**												
Regularly	20 (24.39)	194 (50.26)	1		10 (25.00)	109 (56.19)	1		26 (31.71)	69 (45.39)	1	
Rarely	48 (58.54)	122 (31.61)	**0.26 (0.15–0.46)**	**<0.001**	23 (57.50)	50 (25.77)	**0.20 (0.09–0.45)**	**<0.001**	41 (50.00)	56 (36.84)	**0.51 (0.28–0.94)**	**0.031**
**Periodic oral evaluation (month/time)**												
6 months /time	31 (37.80)	101 (26.17)	1		16 (40.00)	30 (15.46)	1		33 (40.24)	53 (34.87)	1	
12 months /time	26 (31.71)	110 (28.50)	1.30 (0.72–2.34)	0.383	12 (30.00)	60 (30.93)	**2.67 (1.12–6.35)**	**0.027**	22 (26.83)	42 (27.63)	1.19 (0.61–2.33)	0.615
When the child has a toothache	25 (30.49)	175 (45.34)	**2.15 (1.20–3.84)**	**0.010**	12 (30.00)	104 (53.61)	**4.62 (1.97–10.83)**	**<0.001**	27 (32.93)	57 (37.50)	1.31 (0.70–2.47)	0.396

(Univariate regression analysis: the difference is statistically significant with *p* < 0.05. The numbers in bold indicate a statistically significant difference).

**Table 3:** Multivariate regression analysis on total study subjects (*n* = 468).

Related Factors		Beta Coefficient(B)	OR (95% CI)	*p*-Value
Like soft drinks	No		1	
	Yes	1.21	**3.35 (1.27–5.61)**	**<0.001**
Like eating sweet candy and cake	No		1	
	Yes	−3.25	**0.40 (0.11–0.81)**	**<0.001**
Sticky sweet cake	No		1	
	Yes	2.58	**13.20 (9.98–15.66)**	**<0.001**
Sweet marshmallows	No		1	
	Yes	1.15	**3.16 (2.02–5.91)**	**<0.001**
Periodic oral evaluation	6 months/time		1	
	12 months/time	0.66	1.92 (0.59–6.28)	0.267
	When has toothache	1.37	**3.93 (1.44–6.02)**	**<0.001**
Need to treat ECC	No		1	
	Yes	1.14	**3.13 (1.72–4.25)**	**<0.001**

(Multivariate regression analysis: the difference is statistically significant with *p* < 0.05. The numbers in bold indicate a statistically significant difference).

**Table 4:** Multivariate regression analysis in the Obese and Normal Groups.

Related Factors		Obese Group (*n* = 234)			Normal Group (*n* = 234)		
		Beta Coefficient(B)	OR (95% CI)	*p*-Value	B	OR (95% CI)	*p*-Value
Like soft drinks	No		1				
	Yes	1.42	**4.14 (2.99–5.64)**	**<0.001**			
Sweet marshmallows	No		1				
	Yes	2.53	**12.55 (7.07–18.11)**	**<0.001**			
Drink milk at night	No						
	Yes	1.44	**4.22 (2.96–6.03)**	**<0.001**			
Sticky sweet cake	No						
	Yes				2.72	**15.22 (3.90–59.48)**	**<0.001**
Hard lollipop	No				−2.19		
	Yes				−2.19	**0.11 (0.03–0.37)**	**<0.001**
Clean teeth after snacking	No						
	Yes				−1.9	**0.15 (0.05–0.50)**	**0.002**

(Multivariate regression analysis: the difference is statistically significant with *p* < 0.05. The numbers in bold indicate a statistically significant difference).

## Data Availability

Data is contained within the article.

## Abbreviations

ECC: childhood caries; S-ECC: severe early childhood caries; DMFT: decayed, missing, and filled teeth; DMFS: decayed, missing, and filled surfaces.
